# β2-Adrenergic Receptor-Mediated HIF-1α Upregulation Mediates Blood Brain Barrier Damage in Acute Cerebral Ischemia

**DOI:** 10.3389/fnmol.2017.00257

**Published:** 2017-08-14

**Authors:** Yanyun Sun, Xi Chen, Xinyu Zhang, Xianzhi Shen, Mengwei Wang, Xiaona Wang, Wen-Cao Liu, Chun-Feng Liu, Jie Liu, Wenlan Liu, Xinchun Jin

**Affiliations:** ^1^Jiangsu Key Laboratory of Translational Research and Therapy for Neuro-Psycho-Diseases and Institute of Neuroscience, Department of Neurology, The Second Affiliated Hospital of Soochow University Suzhou, China; ^2^School of Pharmacy, Key Laboratory of Molecular Pharmacology and Drug Evaluation, Yantai University, Ministry of Education Yantai, China; ^3^The People’s Hospital of Baoan Shenzhen Shenzhen, China; ^4^Department of Emergency, Shanxi Provincial People’s Hospital Taiyuan, China; ^5^Translational Center for Stem Cell Research, Tongji Hospital, Stem Cell Research Center, Tongji University School of Medicine Shanghai, China; ^6^The Central Laboratory, Shenzhen Second People’s Hospital, Stem Cell Research Center, The First Affiliated Hospital of Shenzhen University Shenzhen, China

**Keywords:** cerebral ischemia, HIF-1α, β2-AR, blood brain barrier, tight junction proteins, matrix metalloproteinase

## Abstract

Disruption of the blood brain barrier (BBB) within the thrombolytic time window is an antecedent event to intracerebral hemorrhage in ischemic stroke. Our recent studies showed that 2-h cerebral ischemia induced BBB damage in non-infarcted area and secreted matrix metalloproteinase-2 (MMP-2) accounted for this disruption. However, the factors that affect MMP-2 secretion and regulate BBB damage remains unknown. Since hypoxia-inducible factor-1 alpha (HIF-1α) was discovered as a mater regulator in hypoxia, we sought to investigate the roles of HIF-1α in BBB damage as well as the factors regulating HIF-1α expression in the ischemic brain. *in vivo* rat middle cerebral artery occlusion (MCAO) and *in vitro* oxygen glucose deprivation (OGD) models were used to mimic ischemia. Pretreatment with HIF-1α inhibitor YC-1 significantly inhibited 2-h MCAO-induced BBB damage, which was accompanied by suppressed occludin degradation and vascular endothelial growth factor (VEGF) mRNA upregulation. Interestingly, β2-adrenergic receptor (β2-AR) antagonist ICI 118551 attenuated ischemia-induced BBB damage by regulating HIF-1α expression. Double immunostaining showed that HIF-1α was upregulated in ischemic neurons but not in astrocytes andendothelial cells. Of note, HIF-1α inhibition with inhibitor YC-1 or siRNA significantly prevented OGD-induced VEGF upregulation as well as the secretion of VEGF and MMP-2 in neurons. More importantly, blocking β2-AR with ICI 118551 suppressedHIF-1α upregulation in ischemic neurons and attenuated occludin degradation induced by the conditioned media of OGD-treatedneurons. Taken together, blockade of β2-AR-mediated HIF-1α upregulation mediates BBB damage during acute cerebral ischemia. These findings provide new mechanistic understanding of early BBB damage in ischemic stroke and may help reduce thrombolysis-related hemorrhagic complications.

## Introduction

Hemorrhagic transformation (HT), the most serious complication in thrombolytic therapy for acute ischemic stroke (Jin et al., 2015; Wang et al., 2015; Liu H. et al., 2016; Ji et al., 2017), occurs as a result of failure of the blood brain barrier (BBB) integrity (Simard et al., 2007). In the past decade, reperfusion-induced BBB injury has been an important focus probably because most of the damaging consequences of BBB injury (hemorrhage and edema) won’t be presented until the blood flow to the ischemic brain is restored (Simard et al., 2007; Hafez et al., 2014; Shi et al., 2016). Accumulating evidence showed that the brain regions with initial BBB damage often undergo HT during thrombolytic reperfusion (Kastrup et al., 2008; Sun et al., 2010; Leigh et al., 2014) and the importance of early ischemic BBB damage is increasingly recognized and is now emerging as pretreatment predictor as well as a promising target for clinical intervention to reduce thrombolysis-associated HT (Jin et al., 2014; Shi et al., 2016). However, ischemia-induced BBB damage, particularly at the early damage that occurs within the 4.5 h thrombolytic time window (Hacke et al., 2008), remains a much less-well studied topic.

Our recent studies showed that 2 h of ischemia caused brain tissue damage in cortex and BBB damage in non-infarcted ventromedial striatum and preoptic area (Jin et al., 2012; Wang et al., 2016). Ischemia-induced matrix metalloproteinase-2 (MMP-2) secretion lead to disrupted BBB and degradation of occludin (Jin et al., 2012; Liu et al., 2012; Wang et al., 2016), which is a key tight junction protein to seal the gaps between adjacent endothelial cells and thus restrict paracellular permeability of the BBB (Wolburg and Lippoldt, 2002). Occludin degradation is frequently seen in the compromised BBB following cerebral ischemia and reperfusion (Yang et al., 2007; Liu et al., 2012). However, we do not know the factors that affect MMP-2 secretion and regulate BBB damage. Hypoxia-inducible factor-1 alpha (HIF-1α) is constitutively transcribed and translated in most cell types, but the protein has only a half-life of less than 5 min under normoxic conditions (Shi, 2009; Semenza, 2014). It is induced in the brain under hypoxia/ischemia conditions and was discovered as a mater regulator in hypoxia (Bernaudin et al., 2002; Zhang et al., 2014). HIF-1α and downstream vascular endothelial growth factor (VEGF) have been shown to play important roles in ischemia-reperfusion induced BBB damage (Chen et al., 2009, 2010). For example, HIF-1α up-regulates VEGF and leads to BBB disruption (Semenza, 2014), while suppression of HIF-1α and VEGF reduces acute hyperglycemia-induced HT in the ischemic brain (Chen et al., 2010; Zhang Z. et al., 2016). Of note, VEGF secreted by hypoxic Müller cells induced MMP-2 activity in endothelial cells (Rodrigues et al., 2013). However, the role of HIF-1α in BBB damage within the first several hours after stroke onset is not known. More important, factors regulating HIF-1α under ischemia remain to be elucidated.

Activation of β2-adrenergic receptor (β2-AR) has been shown to upregulate HIF-1α in cancer cells (Shan et al., 2013). Ischemia produces a significant increase of norepinephrine (NE) in the striatum (Matsumoto et al., 1993), and NE, in turn, upregulates VEGF and MMP-2 (Yang et al., 2006). Therefore, in this study, we tested the hypothesis on an *in vivo* model of middle cerebral artery occlusion (MCAO) and an *in vitro* model of oxygen glucose deprivation (OGD) that cerebral ischemia induces β2-AR activation, and activated β2-AR upregulates HIF-1α to promote MMP-2 secretion and BBB disruption. Our data showed that β2-adrenergic receptor inhibition attenuated HIF-1α upregulation as well as BBB damage within the first several hours of cerebral ischemia.

## Materials and Methods

### Animal Model of Focal Cerebral Ischemia

Sprague-Dawley rats were purchased from SLAC Company (Shanghai, China). They were housed 2–3 per cage under constant temperature (23 ± 1°C) and light-controlled vivarium (12-h light/12-h dark cycle). Rats housed in the same cage underwent the same manipulations. Food and water were available *ad libitum*. All animal procedures were approved by the University Committee on Animal Care of Soochow University and performed according to the NIH Guide for the Care and Use of Laboratory Animals. All efforts were made to minimize animal suffering and to reduce the number of animals. Rats weighing 270–290 g were subjected to 2-h MCAO using the intraluminal suture occlusion model, as we described previously (Liu et al., 2017). Prior to reperfusion, all rats included in this study displayed typical neurologic deficit of MCAO, circling to the left (non-ischemic side). Detailed animal usage for each experiment was listed in figure legends. Successful MCAO was further confirmed by 2,3,5-triphenyltetrazolium chloride (TTC) staining.

### YC-1 Administration

To establish a causal role of HIF-1α in early ischemic BBB damage, the HIF-1α inhibitor YC-1 (Cayman Chemical Company, Ann Arbor, MI, USA dissolved in a solution of 1% dimethyl sulfoxide, DMSO) or vehicle was administered at 2 mg/kg body weight through femoral vein at 24 h and 30 min prior to the onset of ischemia. To inhibit HIF-1α, researchers have used various dosages of YC-1 ranging from 1 mg/kg to 30 mg/kg body weight (Hsiao et al., 2004; Yeh et al., 2007). We chose the dose based on previous publications (Fischer et al., 2002; Schoch et al., 2002; Yan et al., 2011).

### ICI 118551 Administration

To block interaction of NE with β2-AR, β2-AR antagonist ICI 118551 (30 nmol/μL, 0.5 mL, Sigma) or vehicle was injected into the ventral striatum of the ischemia hemisphere with a Hamilton syringe at a rate of 0.25 μL/min 10 min before MCAO. The coordinates for ventral striatum are taken from the atlas of Paxinos and Watson (1986) (AP, −1.0 mm; ML, −2.0 mm; DV, −7.0 mm).

### Evan’s Blue Leakage Detection

Immediately after 2-h MCAO, Evan’s blue dye (EB; Sigma, St. Louis, MO, USA, 2% wt/vol in PBS) was intravenously administered (3 mL/kg) via the left femoral vein. All rats were reperfused for 10 min to allow sufficient circulation of EB to the ischemic brain, but minimize the impact of reperfusion on BBB integrity. At the end of reperfusion, the rat was transcardially perfused with ice-cold PBS and then the brain was quickly taken out.

1. EB leakage was used to check the spatial distribution of BBB damage. Ten consecutive 1-mm-thick coronary slices were sectioned from a 10-mm-thick brain region which was 3 mm away from the tip of the frontal lobe. Besides characterizing the topographic distribution of EB leakage, the mean leakage area was calculated as averaged area proportion of the sections measured.
2. EB leakage was used to detect the co-localization of BBB damage and occludin. Twenty-micrometer-thick cryosection was cut from the 8-mm-thick brain region as we described (Jin et al., 2012) and mounted for fluorescence microscopy observation. BBB disruption was visualized as leakage of EB, which appeared as red fluorescence on brain sections. Brain sections were subjected to immunostain analysis for occludin expression. The experimental procedures for immunostain were described in details below.
3. EB was infused via femoral vein and its content in ischemic hemisphere compared to contralateral hemisphere to quantify BBB disruption as previously reported (Liu et al., 2017). Briefly, rat brain was quickly removed after transcardial perfusion with PBS. Non-ischemic and ischemic hemispheric tissues were harvested as described above and homogenized in 50% wt/vol trichloroacetic acid (Sigma, St. Louis, MO, USA). After centrifugation, the supernatant was diluted four folds with ethanol, and fluorescence intensity (μg/mL) was measured on a microplate fluorescence reader (Infinite M200 Pro; TECAN, Austria). The total EB content (μg) in each sample was derived from concentrations of external standards (1–20 μg/mL). The difference of dye content between ischemic and nonischemic (NI) hemispheric tissue was calculated as EB leakage and expressed as per gram of brain tissue (μg/g).

### Immunostaining

Immediately after 2-h MCAO and 10 min reperfusion, the rat was perfused with ice-cold PBS followed by 4% PFA. The 20-μm-thick cryosection was used for immunostaining analysis for occludin, HIF-1α and VEGF as we described previously (Wang et al., 2017). In brief, non-specific binding sites were blocked by pre-incubating tissue for 1 h at room temperature in PBS containing 0.1% Triton X-100, 1% BSA, and 5% goat serum. Sections were then incubated overnight with anti-HIF-1α (1:200 dilution, Novus), anti-VEGF antibody (1:200 dilution, Abcam), anti-NeuN (1:200 dilution, Millipore), anti-GFAP (1:2000 dilution, Millipore), anti-RECA-1 (1:100 dilution, Abcam) or anti-occludin (1:150 dilution, Invitrogen) primary antibody at 4°C. The latter was followed by incubation with Cy3 conjugated secondary antibody (anti-mouse, 1:800 dilution) or 488-conjugated secondary antibody (anti-rabbit, 1:800 dilution) for 2 h at room temperature. Immunostain was visualized under LSM 700 confocal laser-scanning microscope (Zeiss), and images were taken from the ischemic region and the mirrored region on the NI hemisphere.

### Cell Culture

Mouse brain microvascular endothelial cells bEND3 (American Type Culture Collection) were grown as a monolayer in DMEM with 10% fetal bovine serum (FBS), 100 U/ml penicillin and 100 μg/mL streptomycin at 37°C in a humidified incubator with 5% CO_2_ and 95% room air. The cells were subcultured into 60 mm dishes coated with type I collagen (Nakamuta et al., 2005) and allowed to grow to confluence before exposure to OGD for 2 h. After OGD treatment (3-(4,5-dimethylthiazole-2-yl)-2,5-diphenyl tetrazolium, MTT; Sigma) assay was used to assess the cytotoxicity.

SH-SY5Y cells (neurons) were purchased from American Type Culture and was cultured in DMEM/1640 (1:1; Gibico, Life technologies) containing 10% FBS, 100U/mL penicillin and 100 μg/mL streptomycin, respectively, at 37°C in a humidified incubators with 5% CO_2_ and 95% room air. The cells were subcultured into 6-well cell culture cluster and allowed to grow to 80%–90% confluence before exposure to OGD for 2 h.

Primary astrocyte cultures were prepared from 2-day-old neonatal C57BL/6J mice. In brief, dissociated cortical cells were suspended in DMEM/1640 (Gibico, Life Technology) containing 25 mM glucose, 4 mM glutamine, 1 mM sodium pyruvate, and 10% FBS and plated on uncoated 25 cm^2^ flasks at a density of 6 × 10^5^ cells cm^−2^. Monolayers of type 1 astrocytes were obtained 12–14 days after plating. Non-astrocytic cells were detached from the flasks by shaking and removed by changing the medium. Astrocytes were dissociated by trypsinization and then reseeded on uncoated T75 flasks. These cells were allowed to grow to 80%–90% confluence before exposure to 2-h OGD treatment.

### OGD Treatment

To mimic acute ischemia-like conditions *in vitro*, SH-SY5Y cells were exposed to OGD for 2 h as we described previously (Liu J. et al., 2016). In brief, confluent SH-SY5Y cells were subjected to an ischemic injury by transferring cultures to glucose free medium (DMEM/1640 without glucose) pre-equilibrated with 95% N_2_ and 5% CO_2_. Cells were then incubated in a humidified airtight chamber (MIC-101, Billups-Rothberg Inc., Del Mar, CA, USA) equipped with an air lock and flushed with 5% CO_2_/95% N_2_ for 15 min. The chamber was then sealed and kept at 37°C for another 105 min. Control cultures were incubated with normal DMEM/1640 medium without FBS for 2 h at 37°C in 5% CO_2_/95% air. Immediately after OGD treatment, the conditioned media (CM) and cells were collected separately for further analyses.

### YC-1 and ICI 118551 Treatment for *In Vitro* Study

Cells grown on six-well plates at 80%–90% confluence were incubated with the corresponding medium containing HIF-1α inhibitor YC-1 (10 μmol/L) at 2 h before OGD or β2-AR antagonist ICI 118551 (1 μmol/L) at 10 min before OGD.

### Occludin Degradation by Conditioned Media from OGD-Neurons

At 24 h after seeding, the media were replaced with conditioned media collected from OGD-treated neurons (OGD-neuron CM) or media from control neurons without OGD treatment (Neuron media). Endothelial cells that were maintained in regular endothelial cell media (Media) served as controls. To investigate whether the β2-AR antagonist ICI 118551 suppressed occludin degradation, endothelial cells exposed to OGD-neuron CM were co-treated with vehicle or β2-AR antagonist ICI118 551 at the concentrations of 1 μmol/L.

### siRNA Transfection

SH-SY5Y cells at 60%–70% confluence were transfected with 6 μL HIF-1α siRNA (Santa Cruz, sc-35561) or control siRNA-A (Santa Cruz, sc-37007) which was diluted with the same volume of transfection reagent (Santa Cruz, sc-29528) according to manufacturer’s instruction. Specific silencing was confirmed by western blot.

### Gel Gelatin Zymography

Tissue were homogenized in MMP lysis buffer (50 mM Tris-HCl PH 7.6, 150 mM NaCl, 5 mM CaCl_2_•2H_2_O, 0.05% Brij-35, 0.02% NaN_3_, 1% Triton X-100) and MMP-2/9 levels in homogenates were assessed by gel gelatin zymography as we described previously (Shu et al., 2015).

### Western Blot Analysis for Occludin, HIF-1α and VEGF

Homogenate aliquots (30 μg of total protein) were boiled and then electrophoresed in 10% SDS-PAGE acrylamide gels, transferred onto nitrocellulose membranes (Bio-Rad) and incubated for 1 h in Tris-buffered saline and 0.1% Tween 20 (TBS-T) containing 5% nonfat milk. Membranes were then incubated overnight at 4°C with primary antibodies against occludin (1:300, Invitrogen), HIF-1α (1:300, Novus), VEGF (1:500, Abcam), washed in TBS-T, and then incubated for 2 h at room temperature with corresponding HRP-conjugated anti-rabbit or anti-mouse antibodies (1:3000, Boster). The membranes were developed with the SuperSignal West Pico HRP substrate kit (Pierce) and photographed on a Gel DOC^TM^ XR^+^ image station (Bio-rad). Protein band intensities were quantitated after normalization to β-actin or total protein stained by Ponceau S.

### Real-Time RT-PCR

Total cellular RNA was isolated using Trizol reagents (Invitrogen) according to manufacturer’s protocol as we described previously (Jin et al., 2015). RNA (0.5 μg) was reverse-transcribed (RT) with random primers in a 20 μL final reaction volume using TaqMan^®^ Reverse Transcription Kits (Applied Biosystems). 0.5 μL RT products were amplified with the 7900HT Fast Real-Time PCR System (Applied Biosystems) in a 10 μL final reaction volume using SYBR^®^ Green PCR Master Mix (Applied Biosystems) under the following conditions: 2 min at 50°C and 10 min at 95°C, followed by a total of 40 cycles of two temperature cycles (15 s at 95°C and 1 min at 60°C). Primers (Integrated DNA Technologies) for VEGF and glyceraldehydes 3-phosphate dehydrogenase (GAPDH) were designed against known mouse sequences: VEGF forward: 5′-AGAAAGCCCATGAAGT GGTG-3′, reverse: 5′-ACTCCAGGGCTTCATCATTG-3′; β-actin forward: 5′-ACTATCGGCAATGAGCGGTTCC-3′, reverse: 5′-AGCACTGTGTTGGCATAGAGGTC-3′. The fluorescence threshold value (C_t_ value) was calculated using the SDS Enterprise Database software (Applied Biosystems). The relative value of mRNA expression was calculated by the comparative ΔΔDC_t_ method described in our previous publication (Liu et al., 2007). In brief, mean C_t_ values were normalized to the internal control GAPDH and the difference was defined as ΔΔC_t_. The difference between the mean ΔC_t_ values of treated and untreated cells was calculated and defined as ΔΔDC_t_. The comparative mRNA expression level was expressed as 2^−ΔΔDC_t_^.

### ELISA Assay for VEGF

VEGF contents in cell culture supernatants were assessed using an ELISA kit from Boster (Wuhan, China). Aliquots of 100 μL of cell conditioned media were added to the appropriate microtiter wells provided by the kit. For the standard curve, 100 μL of different concentration VEGF standards and the blank standard were added to the appropriate microtiter wells. The plate was then incubated for 1.5 h at 37°C. After discarding the liquid of each well (no wash), 100 μL of biotinylated anti-human VEGF antibody solution was added into each well except the chromogen blanks and incubated for 1 h at 37°C. After washing three times with the wash buffer, 100 μL avidin-peroxidase complex working solution was added to each well, except the chromogen blanks, and incubated for 30 min at 37°C. Lastly, 100 μL of stabilized chromogen was added to each well, and incubated for 30 min at 37°C in the dark before the addition of 100 μL of stop solution. The absorbance at 450 nm was recorded on a plate reader. A standard curve was generated and the concentrations for unknown samples were obtained from the standard curve.

#### Statistical Analysis

The data are presented as mean ± SEM. Statistical analysis was carried out using ANOVA (SPSS software, version 17.0). Significant effects were probed using Newman-Keuls *post hoc* comparison. A value of *P* < 0.05 was considered statistically significant.

## Results

### HIF-1α and VEGF Changes after 2-h MCAO

**Figure 1:**
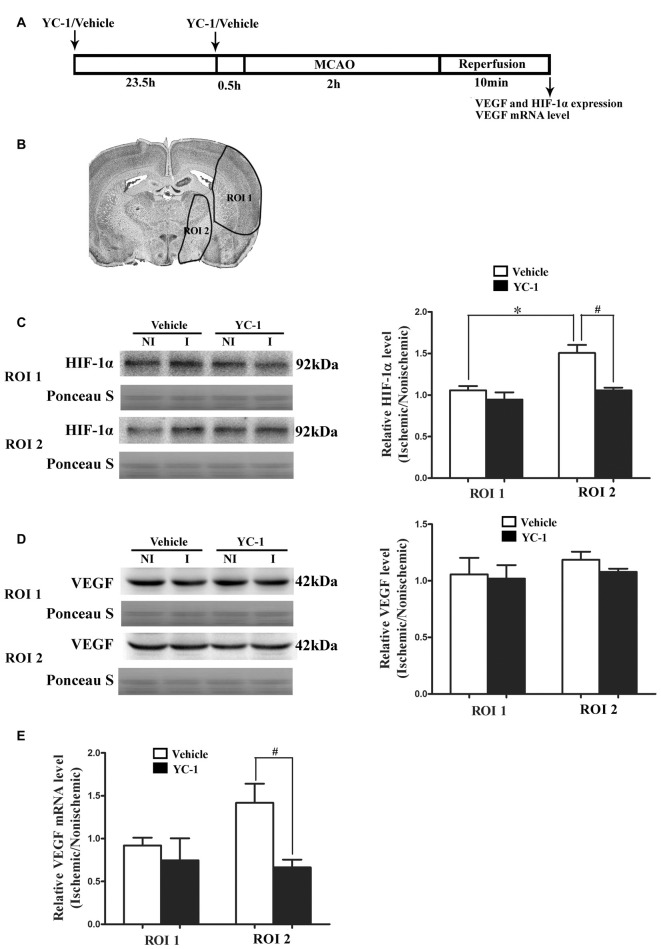
Effect of 2-h ischemia on hypoxia-inducible factor-1 alpha (HIF-1α) and vascular endothelial growth factor (VEGF) expression. **(A)** Diagram of the experimental procedure. **(B)** The black outlines showed the two regions of interest (ROI): 1, parietal and insular cortex and dorsal striatum; 2, ventral striatum and preoptic area. **(C)** Representative western blot images of HIF-1α protein in ROI 1 and 2 in ischemic (I) and nonischemic (NI) hemisphere (left panel). Ratios of HIF-1α (I/NI) were quantitated (right panel). **(D)** Representative western blot images of VEGF protein in region of ROI 1 and 2 in I and NI hemisphere (left panel). Ratios of VEGF (I/NI) were quantitated (right panel). After 2-h middle cerebral artery occlusion (MCAO), there was a significant increase of HIF-1α protein **(C)** but not VEGF protein **(D)** in ROI 2 but not ROI 1 and pretreatment with HIF-1α inhibitor YC-1 significantly prevented this increase. ^#^*P* < 0.05 vs. Vehicle group in ROI 2, *P < 0.05 vs. Vehicle group in ROI 1. **(E)** Ratio of VEGF mRNA (I/NI) was quantitated. Two-hour MCAO induced significant upregulation of VEGF mRNA and pretreatment with YC-1 significantly prevented this increase (^#^*P* < 0.05 vs. Vehicle). Data were expressed as mean ± SEM, *n* = 4/group.

HIF-1α is a key mediator of the adaptive cellular response to hypoxia and VEGF has been implicated in BBB permeability increase (Schoch et al., 2002; Yan et al., 2011). To determine the roles of HIF-1α and VEGF in BBB damage after 2-h MCAO, western blot was used to compare HIF-1α and VEGF levels in the regions of interest (ROI 1, tissue damage area; ROI 2, BBB damage area; Jin et al., 2012). As shown in Figure 1, 2-h MCAO induced a nearly 1.5-fold increase of HIF-1α protein in ROI 2 (*P* < 0.05), but not ROI 1 (*P* > 0.05, Figure 1C). Pretreatment with HIF-1α inhibitor YC-1 at a dose of 2 mg/kg body weight significantly prevented this increase (*P* < 0.05, Figure 1C). Surprisingly, 2-h MCAO did not change the protein levels of VEGF (Figure 1D), but significantly increased VEGF mRNA expression, and YC-1 significantly inhibited VEGF upregulation (Figure 1E). The results suggested that 2-h MCAO induced VEGF mRNA expression in a HIF-1α-dependent manner.

### HIF-1α Inhibitor YC-1 Inhibited 2-h MCAO-Induced BBB Disruption and Occludin Degradation

**Figure 2:**
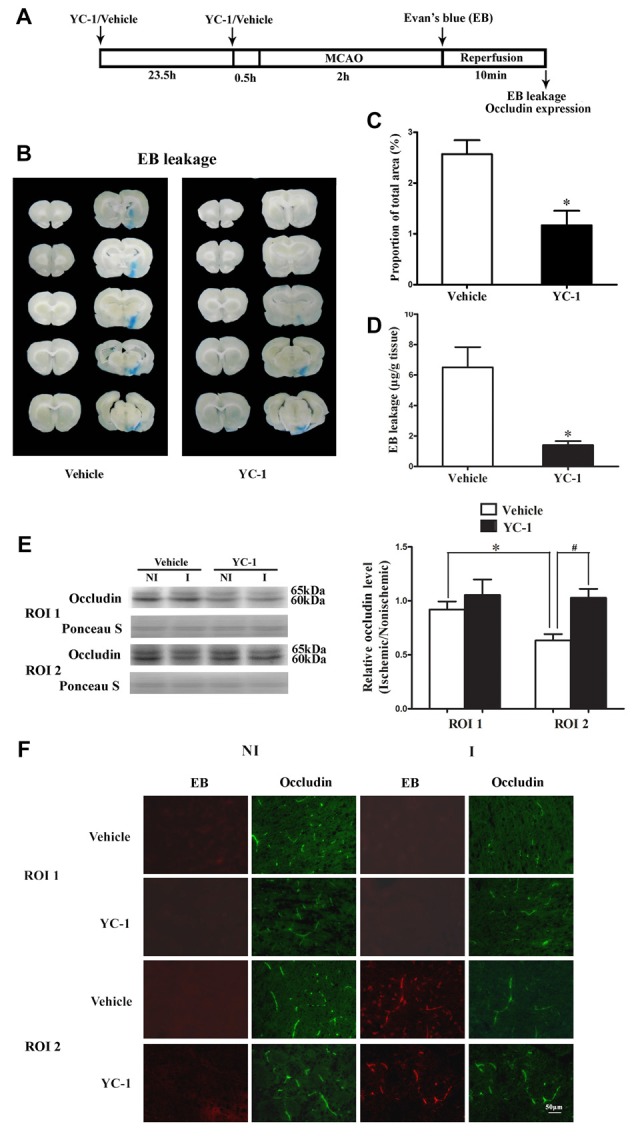
Effect of YC-1 treatment on 2-h ischemia-induced Evan’s blue (EB) leakage and occludin expression. **(A)** Diagram of the experimental procedure. Rats received YC-1 at 24 h and 30 min before ischemia onset. **(B)** Ten consecutive sections showed EB leakage from vehicle or YC-1 treated rats. **(C)** EB leakage was quantitated and expressed as average area proportion of section measured (%). ^#^*P* < 0.05 vs. Vehicle group. *n* = 6/group. **(D)** EB leakage in the brain tissue was quantitated according to the external EB standard curve. EB leakage was expressed as per gram of brain tissue (μg/g). ^#^*P* < 0.05 vs. Vehicle group. Data were expressed as mean ± SEM, *n* = 5/group. **(E)** Western blot was conducted to detect occludin protein levels in the NI and ischemic (I) hemispheric tissue. A representative western blot revealed occludin protein expression in ROI 1 and ROI 2 treated with YC-1 or vehicle (left panel). The band intensity of occludin was quantitated after normalization to the Ponceau S (right panel). Two-hour MCAO induced a significant reduction in occludin protein level in ROI 2 (**E**, right panel. *P < 0.05 vs. Vehicle group in ROI 1), but not in ROI 1 (*P* > 0.05). Pretreatment with YC-1 attenuated occludin degradation (^#^*P* < 0.05 vs. Vehicle group). Data were expressed as mean ± SEM, *n* = 5/group. **(F)** EB leakage (red) was seen in the ischemic ROI 2 after 2-h ischemia and IHC showed occludin degradation in the area where EB leakage occurred. Pretreatment with YC-1 significantly attenuated occludin degradation as well as EB leakage. *n* = 3/group. Data were expressed as mean ± SEM.

EB was used as a marker to evaluate BBB integrity (Wang et al., 2017). We next examined the effect of HIF-1α inhibitor YC-1 treatment on 2-h MCAO-induced BBB disruption and occludin degradation (Figure 2A). Representative images of EB dye in wet brain tissue was shown in Figure 2B. Obvious EB leakage was observed in the ipsilateral hemisphere of brain from rats subjected to 2-h MCAO (Figure 2B). HIF-1α inhibitor YC-1 treatment dramatically reduced the area of EB leakage (Figure 2C) and quantification data showed that YC-1 decreased ~78% of EB leakage (Figure 2D). These results indicated that HIF-1α inhibition was effective in reducing BBB damage within the first 2 h after ischemia onset.

Our recent study showed that loss of occludin contributed to BBB damage after 2-h ischemia (Wang et al., 2016). To determine whether ischemia-induced HIF-1α upregulation contributed to the rapid disruption of occludin, IHC and western blot were conducted to detect occludin protein levels in ROI with BBB leakage. Our data showed that pretreatment with YC-1 significantly inhibited ischemia-induced occludin degradation in ROI 2 (EB leakage area, Figure 2F). Western blot was done to confirm the IHC results and found that 2-h MCAO induced a significant reduction in occludin protein levels in ROI 2, but not ROI 1 (Figure 2E). These results indicated that HIF-1α inhibition significantly attenuated occludin degradation within the first 2 h after ischemia onset (Figure 2E).

### Effect of β2-AR Antagonist ICI 118551 on Ischemia-Induced HIF-1α Upregulation and BBB Disruption

**Figure 3:**
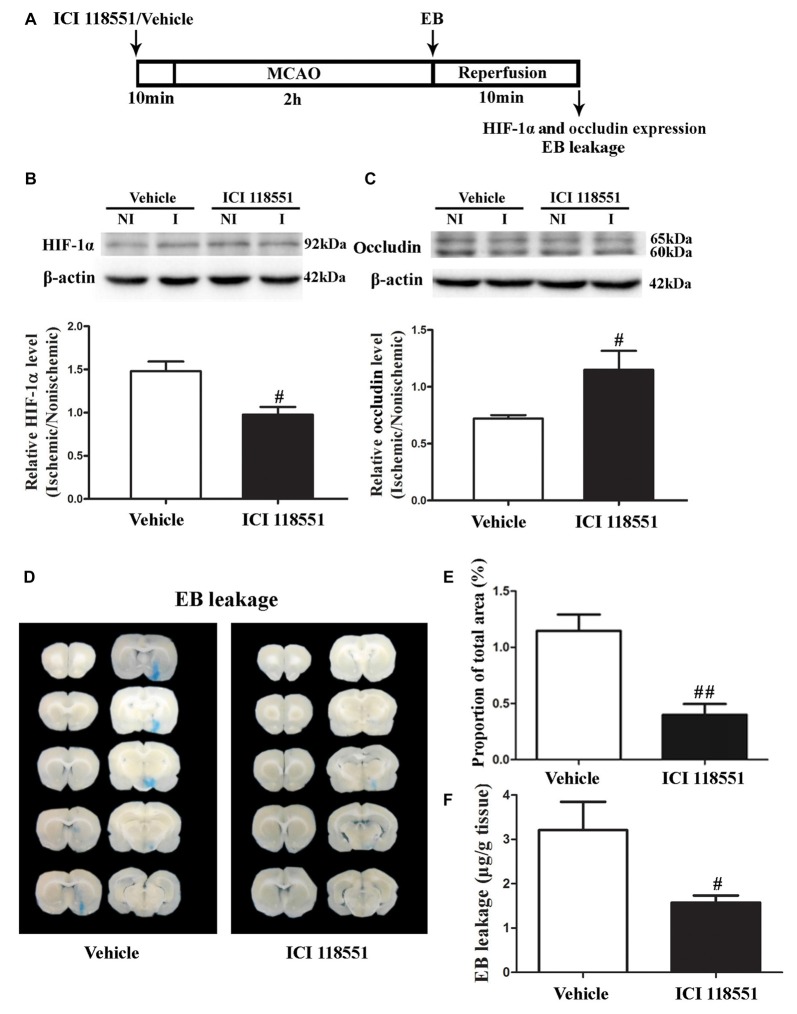
Effect of β2 adrenergic receptor (β2-AR) antagonist ICI118551 on 2-h ischemia-induced HIF-1α expression and blood brain barrier (BBB) disruption. **(A)** Diagram of the experimental procedure. Rats received ICI118551 10 min before the onset of ischemia. **(B,C)** Western blot was conducted to detect HIF-1α **(B)** and occludin **(C)** protein levels in the NI and ischemic (I) hemispheric tissue. A representative western blot revealed HIF-1α **(B)** and occludin **(C)** protein expression treated with YC-1 or vehicle. The band intensity of HIF-1α and occludin was quantitated after normalization to the β-actin. Two-h MCAO induced a significant increase of HIF-1α **(B)** and reduction of occludin **(C)**. Pretreatment with ICI118551 inhibited HIF-1α upregulation and attenuated occludin degradation (^#^*P* < 0.05 vs. Vehicle group). Data were expressed as mean ± SEM, *n* = 5/group. **(D)** Ten consecutive sections showed EB leakage from vehicle or YC-1 treated rats. **(E)** EB leakage was quantitated and expressed as average area proportion of section measured (%). ^##^*P* < 0.01 vs. Vehicle group, *n* = 6/group. **(F)** EB leakage in the brain tissue was quantitated according to the external EB standard curve. EB leakage was expressed as per gram of brain tissue (μg/g). ^#^*P* < 0.05 vs. Vehicle group. Data were expressed as mean ± SEM, *n* = 5/group.

β2-AR has been shown to regulate HIF-1α expression in cancer cells (Shan et al., 2013). To investigate the effect of blocking β2-AR on ischemia-induced HIF-1α upregulation and BBB disruption, rats received ICI118551 (30 nmol/mL, 0.5 mL; Qu et al., 2008) 10 min before MCAO (Figure 3A). Our results showed that blocking β2-AR significantly inhibited 2-h ischemia-induced HIF-1α upregulation (Figure 3B, *P* < 0.05 vs. Vehicle), occludin degradation (Figure 3C, *P* < 0.05 vs. Vehicle) and BBB damage (Figures 3D–F, *P* < 0.05 vs. Vehicle), suggesting that blocking β2-AR inhibited BBB damage through regulating HIF-1α.

### Colocalization of HIF-1α with Endothelial Cells, Neurons and Astrocytes

**Figure 4:**
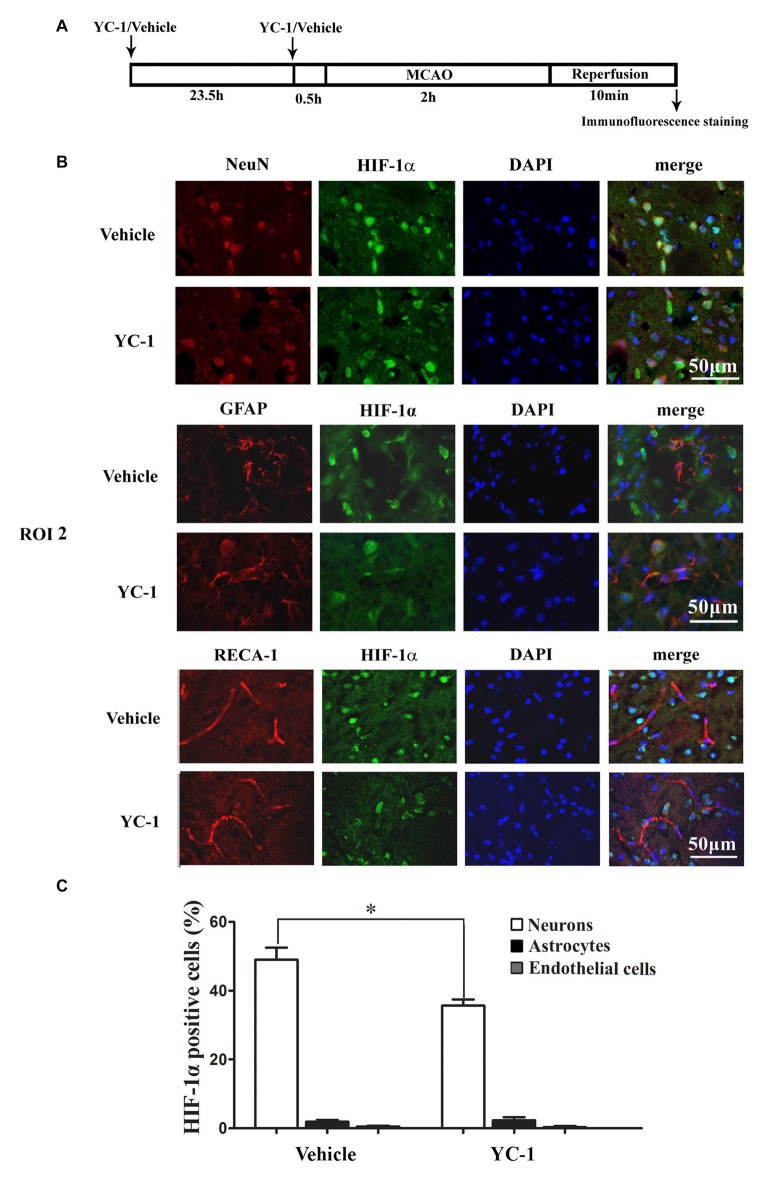
Effect of YC-1 on 2-h ischemia-induced HIF-1α expression in endothelial cells, neurons and astrocytes. **(A)** Diagram of the experimental procedure. Rats received YC-1 at 24 h and 30 min before ischemia onset. The cellular pattern of HIF-1α expression was analyzed by double-immunofluorescence labeling. Double immunostain of HIF-1α (green) and NeuN (marker of neurons, red) showed a good colocalization of HIF-1α and neurons (**B**, upper panel). Double immunostain of HIF-1α (green) and GFAP (marker of astrocytes, red) showed no co-localization of HIF-1α and astrocytes (**B**, middle panel). Double immunostain of HIF-1α (green) and RECA-1 (marker of endothelial cells, red) showed no co-localization of HIF-1α and endothelial cells (**B**, bottom panel). *n* = 3/group. Scale bar = 50 μm. **(C)** YC-1 significantly decreased the proportion of HIF-1α-positive neurons but not astrocytes or endothelial cells *P < 0.05 vs. Vehicle group. *n* = 3/group.

Cell type-specific action for HIF-1α has been defined in astrocytes and neurons in a rat neonatal stroke model (Mu et al., 2003; Vangeison et al., 2008). However, the cellular pattern of HIF-1α expression was not known in the ischemic brain at 2 h after MCAO onset. Double-immunofluorescence labeling was used to determine the cellular localization of HIF-1α, and DAPI staining was conducted to verify the subcellular localization of HIF-1α. As shown in Figure 4, HIF-1α was mainly expressed in neurons (Figure 4B, upper panel), but not in astrocytes (Figure 4B, middle panel) or endothelial cells (Figure 4B, bottom panel) in the ROI 2 at 2 h after MCAO onset. YC-1 significantly decreased the number of HIF-1α-positive neurons, but had no effect on HIF-1α expression in astrocytes or endothelial cells (Figure 4C). These results indicated that the neurons might be major cellular source of HIF-1α early after cerebral ischemia onset.

The cellular distribution and expression of VEGF protein was determined by immunofluorescence with a polyclonal VEGF antibody. VEGF-positive cells were identified to be neurons (colocalization with NeuN but not with GFAP, data not shown).

### Effect of HIF-1α Inhibition on VEGF mRNA Transcription and Protein Secretion

Hypoxia rapidly increased the HIF-1α as well as the mRNA levels of VEGF (Bernaudin et al., 2002), but VEGF protein increase was much later, which occurred at 4 h or 8 h during the phase of reperfusion (Mu et al., 2003). We applied oxygen-glucose deprivation (OGD) to mimic ischemia *in vitro* (Zhang Y. et al., 2016). Human dopaminergic neurons (SH-SY5Y cells) were subjected to 2-h OGD. YC-1 or HIF-1α siRNA was applied to inhibit HIF-1α level and non-targeting siRNA was used as a control siRNA (Ctrl siR). ELISA and western blot were recruited to detect VEGF level in cultured media (CM) and cellular extracts (CE), respectively.

**Figure 5:**
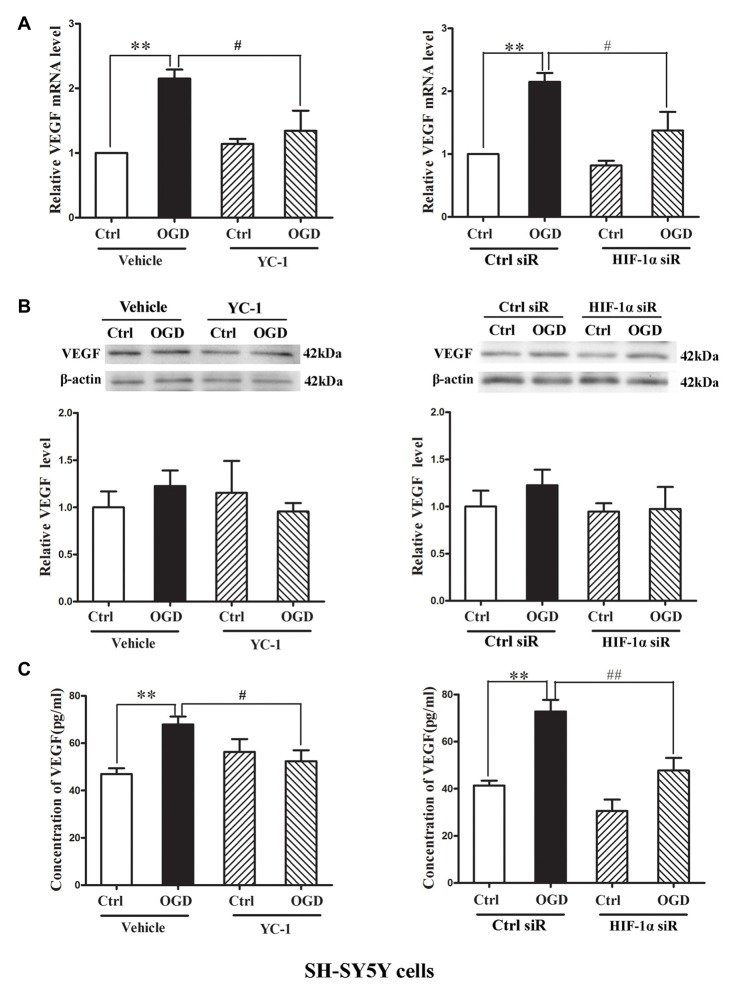
Effect of HIF-1α inhibition by YC-1 or HIF-1α siRNA on 2-h oxygen glucose deprivation (OGD)-induced VEGF secretion in neurons. The neurons were exposed to OGD for 2 h and VEGF secretion was assessed by measuring its content in cellular extracts (CE) and conditioned media (CM) using western blot and ELISA respectively. Two-hours OGD induced a significant increase in VEGF mRNA expression in neurons and pretreatment with YC-1 (**A**, left panel) or HIF-1α siR (**A**, right panel) significantly inhibited this effect. **P < 0.01 vs. control (Ctrl), ^#^*P* < 0.05 vs. OGD, *n* = 4/group. **(B)** Representative blots for VEGF and β-actin (upper panel). The relative VEGF protein level was calculated after normalization to β-actin (bottom panel). Two-hours OGD had no effect on the VEGF protein levels in neurons but induced a significant increase of VEGF in cultured media and pretreatment with HIF-1α inhibitor YC-1 (**C**, left panel) or HIF-1α siR (**C**, right panel) attenuated this VEGF secretion induced by 2-h OGD. **P < 0.01 vs. Ctrl. ^#^*P* < 0.05 vs. OGD. ^##^*P* < 0.01 vs. OGD. HIF-1α siR represents HIF-1α siRNA. Data are expressed as mean ± SEM, *n* = 3/group.

Two-hour OGD induced a significant increase of VEGF mRNA expression in neurons (*P* < 0.05 vs. Ctrl), and pretreatment with YC-1 (Figure 5A, left panel) or HIF-1α siR (Figure 5A, right panel) significantly inhibited this effect (*P* < 0.05 vs. OGD). However, 2-h OGD had no effect on the protein levels of VEGF in the CE (Figure 5B), but significantly increased its levels in the CM (Figure 5C, *P* < 0.05 vs. Ctrl). Pretreatment with YC-1 (Figure 5C, left panel) or HIF-1α siR (Figure 5C, right panel) significantly inhibited VEGF increase in the CM (*P* < 0.05 vs. OGD) suggesting that VEGF secretion was mediated by HIF-1α.

### Effect of HIF-1α Inhibition on MMP-2 Secretion in Neurons and Endothelial Cells

**Figure 6:**
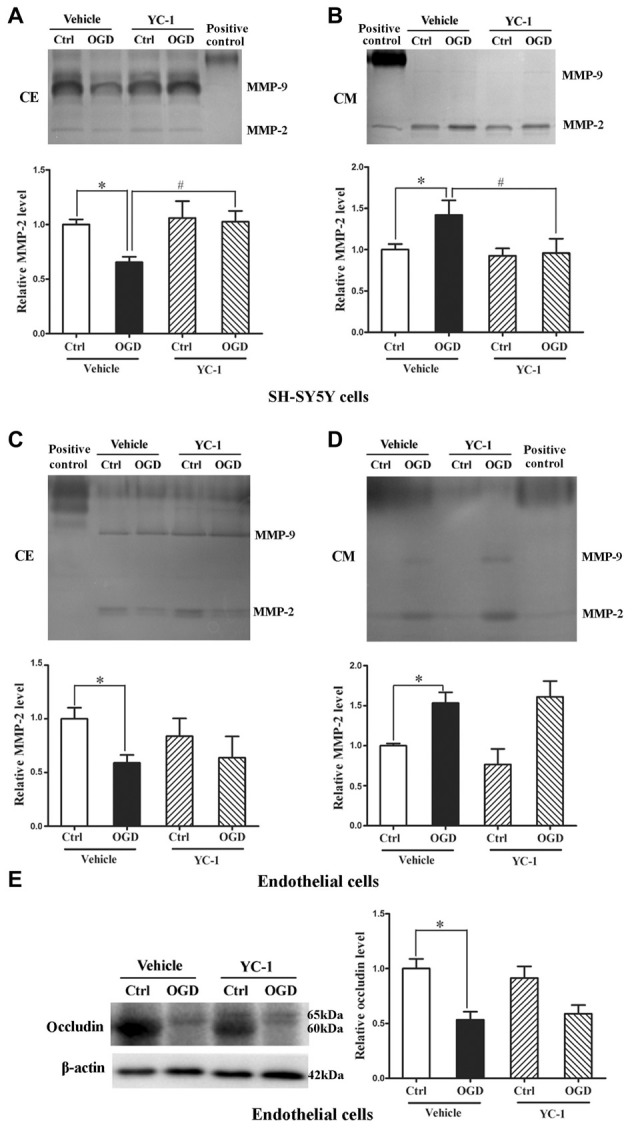
Effect of YC-1 on ischemia-induced matrix metalloproteinase-2 (MMP-2) secretion in neurons and endothelial cells. Gelatin zymography results showed that 2-h OGD elevated MMP-2 levels in conditioned media (CM), which was accompanied by a significant reduction in their levels in whole cellular extracts (CE). **(A)** Representative gelatin zymogram (upper panel) showing that OGD decreased MMP-2 levels in CE of SH-SY5Y cells and YC-1 treatment prevents this decrease. The relative levels of MMP-2 were quantitated (bottom panel). **(B)** Representative gelatin zymogram (upper panel) showing that OGD increased MMP-2 levels in CM of SH-SY5Y cells and YC-1 treatment prevents this increase. The relative levels of MMP-2 were quantitated (bottom panel). **(C)** Representative gelatin zymogram (upper panel) showing that YC-1 treatment did not significantly inhibited OGD-induced MMP-2 decrease in CE of endothelial cells. The relative levels of MMP-2 were quantitated (bottom panel). **(D)** Representative gelatin zymogram (upper panel) showing that YC-1 treatment did not inhibit OGD-induced MMP-2 increase in CM of endothelial cells. The relative levels of MMP-2 were quantitated (bottom panel). **(E)** Pretreatment with ICI118551 did not prevent 2-h OGD-induced occludin degradation in endothelial cells. *P < 0.05 vs. control (Ctrl), ^#^*P* < 0.05 vs. OGD. Data are expressed as mean ± SEM, *n* = 6/group.

We applied *in vitro* cultured brain microvascular endothelial monolayer to mimic the BBB as bEND3 cell line has the ability to maintain BBB characteristics over many passages, is easy to grow and can form functional barriers (Camós and Mallolas, 2010). We have shown that OGD induces MMP-2 secretion in neurons, astrocytes and endothelial cells and the secreted MMP-2 disrupts BBB by degrading occludin (Liu et al., 2012), here we explored the effect of YC-1 on MMP-2 secretion in neurons and endothelial cells. After 2-h OGD, MMP-2 levels in CM and CE were detected by gel gelatin zymography. As shown in Figure 6, OGD-induced MMP-2 increase in the conditioned media accompanied by a decrease in the CE, indicating an OGD-induced MMP-2 secretion in neurons. Pretreatment with YC-1 significantly inhibited MMP-2 secretion in neurons (Figures 6A,B, *P* < 0.05 vs. OGD), but not in endothelial cells (Figures 6C,D). Of note, YC-1 did not reverse OGD-induced occludin degradation in endothelial cells (Figure 6E). These results suggested that HIF-1α mediated OGD-induced MMP-2 secretion in neurons but not in endothelial cells, and further studies are warranted to define the mechanisms underlying MMP-2 secretion in endothelial cells under OGD conditions.

### Effect of β2-AR Antagonist ICI 118551 on Ischemia-Induced HIF-1α Upregulation in Neurons and Endothelial Cells

**Figure 7:**
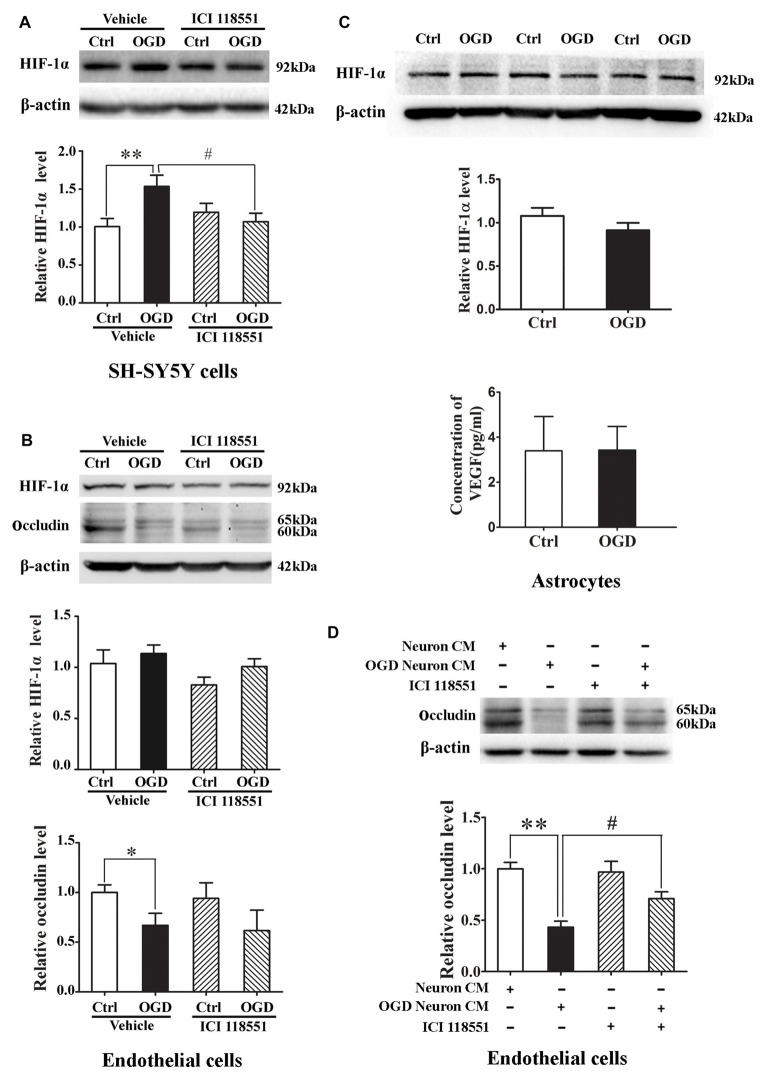
Effect of ICI118551 on ischemia-induced HIF-1α expression and occludin degradation induced by the conditioned media of OGD-treated neurons. HIF-1α expression in neurons, endothelial cells and astrocytes were detected after 2-h OGD. Two-hour OGD induced a significant HIF-1α upregulation in neurons **(A)** but not endothelial cells **(B)** or astrocytes **(C)**. Pretreatment with ICI118551 inhibited OGD-induced HIF-1α upregulation in neurons **(A)**, but did not attenuated OGD-induced occludin degradation in endothelial cells **(B)** *P < 0.05 vs. control (Ctrl), **P < 0.01 vs. control (Ctrl), ^#^*P* < 0.05 vs. OGD. **(D)** Western blot analysis showed that conditioned media (CM) from OGD-treated neurons induced occludin degradation in endothelial cells and pretreatment neurons with ICI 118551 attenuated this degradation. **P < 0.01 vs. neuron CM (Ctrl), ^#^*P* < 0.05 vs. neuron CM.

We next examined the effect of blocking β2-AR on HIF-1α expression in neurons and endothelial cells subjected to 2-h OGD. Neurons or endothelial cells were incubated with ICI 118551 for 10 min before exposing to 2-h OGD. As shown in Figure 7, 2-h OGD significantly upregulated HIF-1α in neurons (Figure 7A), but not in endothelial cells (Figure 7B, upper and middle panel) and astrocytes (Figure 7C, upper and middle panel). Of note, 2-h OGD did not induce VEGF secretion in astrocytes (Figure 7C, bottom panel). Although pretreatment with ICI 118551 did not inhibit OGD-induced occludin degradation in endothelial cells (Figure 7B, upper and bottom panel), it significantly inhibited OGD-induced HIF-1α upregulation in neurons (Figure 7A, *P* < 0.05 vs. OGD). These results suggested that blocking β2-AR inhibited OGD-induced HIF-1α upregulation in neurons.

### Effect of Blocking β2-AR on Occludin Degradation Induced by the Conditioned Media of OGD-Treated Neurons

The above results have demonstrated that OGD induced the secretion of MMP-2 and VEGF into extracellular medium through activating β2-AR-HIF-1α pathway, which mediated occludin degradation in endothelial cells. To further clarify the interaction between neurons and endothelial cells, we exposed endothelial cells to the conditioned media from OGD-treated neurons for 2 h. Western blot analysis showed that the OGD-neuron conditioned media induced occludin degradation in endothelial cells and pretreatment neurons with ICI 118551 inhibited the degradation (Figure 7D).

## Discussion

Tightness of the BBB at the time of thrombolytic intervention is an important factor determining the efficacy of thrombolysis with tPA (Benchenane et al., 2005; Leigh et al., 2014, 2016) and ischemic BBB damage within thrombolytic time window is a promising target (Jin et al., 2014; Liu J. et al., 2016) for clinical intervention to reduce intracranial hemorrhage in patients with intravenous tPA (Leigh et al., 2014) or post-endovascular treatment (Leigh et al., 2016). In our current study, we found that: (1) 2-h ischemia induced BBB damage in non-infarcted ventral striatum accompanied by HIF-1α protein and VEGF mRNA upregulation; (2) HIF-1α inhibition prevented BBB damage by preventing VEGF and MMP-2 secretion in neuron and occludin degradation in endothelial cells; and (3) blocking β2-AR alleviated ischemia-induced BBB damage by reducing HIF-1α level. Taken together, our results provide strong evidence that BBB damage is reduced by blockade of β2-adrenergic receptor-mediated HIF-1α upregulation during acute cerebral ischemia.

Our current results show that 2-h MCAO induces HIF-1α upregulation, which mainly occurs in the non-infarcted ventral striatum and preoptic area, where the brain tissue has been shown to experience moderate cerebral blood flow (CBF) reduction during MCAO (Jin et al., 2012). This finding is consistent with a previous report that HIF-1α was found to be mostly induced in the salvageable tissue in the ischemic brain, a moderate but not prolonged decrease in CBF, induced HIF-1α in the penumbra (Bergeron et al., 1999). We speculate that moderately-reduced CBF may trigger reperfusion-like damaging events such as increased reactive oxygen species generations in hypoperfused tissue to disrupt BBB (Qutub and Popel, 2008). Future studies are warranted to test this hypothesis. It is worth of note, HIF-1α expression in neuron is cell-type-specific in response to hypoxic/ischemic insults, interneurons express a significantly higher level of HIF-1α protein than pyramidal neurons (Ramamoorthy and Shi, 2014) and there are more interneurons than pyramidal neurons in the striatum.

Our data that 2-h ischemia induces HIF-1α upregulation and increased the mRNA of VEGF. This is consistent with a previous study that hypoxia, rapidly increased the nuclear contents of HIF-1α as well as the mRNA levels of VEGF (Bernaudin et al., 2002). Here, we did not observe an increase in VEGF protein levels, which is different from previous reports that VEGF protein is elevated in adult brain in response to stroke (Yeh et al., 2007; Chen et al., 2010; Yan et al., 2011). Lack of prolonged reperfusion in our experimental design may explain this discrepancy, because it has been reported that the protein levels of VEGF after ischemia (without reperfusion) are comparable to the untreated control but are increased two and three folds after 4 h or 8 h of reperfusion, respectively (Mu et al., 2003), indicating that VEGF protein is produced in the reperfusion stage and the ischemia duration may only stimulate the HIF-1α-mediated VEGF mRNA expression, while may not be long enough to induce VEGF protein synthesis. These results are further confirmed by our *in vitro* experiments showing that 2-h OGD induces VEGF mRNA but not protein increase in neurons and pretreatment with either YC-1 or HIF-1α siRNA significantly inhibits this increase. Our *in vitro* data show that there is a significant increase in VEGF protein in the conditioned medium and but does not change VEGF protein contents in the cellular extracts. Our explanation for this finding is that being a secretory protein, the new synthesized VEGF in OGD-treated cells may be secreted into the conditioned media. Therefore, the VEGF protein levels in the cellular extracts are not significantly changed. Our *in vivo* data show that MCAO did not induce VEGF increase in ROI 2 by western blot that measures the total level of VEGF in ischemic brain tissue. Under this experimental condition, we could not accurately separate the extracellular VEGF from intracellular VEGF, as a substantial portion of the extracellular fluid and the soluble proteins would be inevitably lost during the process of protein extraction, and in addition, the amount of extracellular fluid protein in the total amount of tissue protein is very small, or even negligible. From this point of view, our *in vitro* and *in vivo* results of VEGF are consistent.

Our data that HIF-1α is colocalized with the neurons but not astrocytes or endothelial cells indicate that HIF-1α is mainly upregulated in neurons under our experimental conditions. In the brain, the neurons are the most sensitive cell type and robustly respond to hypoxic/ischemic stimulus to upregulate HIF-1α. Unlike many other cells, astrocytes are very resistant to hypoxic stress and severe oxygen deprivation is required to induce HIF-1α signaling and modulate subsequent survival and proliferation in astrocytes (Schmid-Brunclik et al., 2008). Two-hour ischemia may not be strong enough for astrocytes to produce HIF-1α. If the duration of ischemia or reperfusion is extended, astrocytes could then act as an important player for BBB damage. As an example, Li et al. (2014) have reported that 3-h OGD promotes neurons to activate astrocytes to disrupt endothelial barrier via increasing VEGF expression. In addition, cell specific temporal-spatial modulation of HIF-1α signaling pathways has been shown to be a key determinant of functional outcome (Ogunshola and Al-Ahmad, 2012). Understanding cell-type dependent effects of HIF-1α will undoubtedly shed new lights on its role in cerebral ischemia and thus provides potential approaches to augment its beneficial effect and reduce its detrimental function (Engelhardt et al., 2014).

Our previous finding showed that BBB damage is mediated by secreted but not new synthesized MMP-2 at a very stage of ischemia (Liu et al., 2012) has led us to investigate the factors that induces MMP-2 secretion. HIF-1α is well implicated in the control of MMPs that critically contribute to vascular permeability increase under various conditions (Liu and Rosenberg, 2005; Yang et al., 2007; Jin et al., 2013). Here, our data show that OGD induces HIF-1α-dependent MMP-2 secretion in neurons and HIF-1α-independent MMP-2 secretion in endothelial cells. Therefore, when endothelial cells are exposed to OGD, the increased MMP-2 secretion can lead to occludin degradation, and this process is independently of HIF-1α or neurons. However, during MCAO, affected by surrounding cells and the extracellular matrix, endothelial cells may behave differently in the ischemic brain compared to the OGD model. Owing to its high sensitivity to OGD or ischemia, the neurons may be the first cell type responding to ischemic stress and secreting MMP-2 and VEGF in a HIF-1α dependent manner. The secreted MMP-2 and VEGF may then affect the activity of endothelial cells to secret more MMP-2 and VEGF. Therefore, in the MCAO model, we can’t exclude the possibility that the involvement of endothelial cells in BBB disruption may also be dependent on HIF-1α or neurons.

It has been shown that β2-AR-HIF-1α axis played an important role for stress-induced pancreatic tumor growth and angiogenesis (Shan et al., 2013) as well as in NNK-induced pancreatic cancer progression (Zhang et al., 2015). In addition, β2-AR antagonist propranolol has been shown to regress infantile hemangiomas in a HIF-1α-dependent manner. More important, MMP-2 has been shown to mediate β-AR-induced apoptosis in ventricular myocytes (Menon et al., 2005) and cardiac hypertrophy in rats (Miura et al., 2003). Here our data showed that blocking β2-AR significantly inhibits ischemia-induced BBB damage through suppressing HIF-1α expression and occludin degradation, indicating that β2-AR-HIF-1α signaling may represent promising therapeutic targets for preventing ischemia-induced BBB damage.

In summary, BBB damage is reduced by blockade of β2-adrenergic receptor-mediated HIF-1α upregulation during acute cerebral ischemia. The findings may provide new ideas to protect the BBB against ischemic damage and to extend the time window of thrombolysis and reduce cerebral hemorrhage.

## Author Contributions

The work was performed and accomplished by all authors. YS, XC, XZ, XS, MW and XW contributed to the execution of the entire project and the statistical analyses. W-CL, JL, WL and C-FL provided advice on experimental design and interpretation, and comments on the manuscript. YS, XC and XJ wrote the manuscript. All authors have read and approved the final manuscript.

## Conflict of Interest Statement

The authors declare that the research was conducted in the absence of any commercial or financial relationships that could be construed as a potential conflict of interest.

## Abbreviations

BBB: blood brain barrier
β2-AR: β2-adrenergic receptor
CBF: cerebral blood flow
CE: cellular extract
CM: conditioned media
EB: Evan’s blue dye
HIF-1α: hypoxia-inducible factor-1 alpha
HT: hemorrhagic transformation
MCAO: middle cerebral artery occlusion
MMP: matrix metalloproteinase
NE: norepinephrine
OGD: oxygen glucose deprivation
ROI: region of interest
TTC: 2,3,5-triphenyltetrazolium chloride
VEGF: vascular endothelial growth factor.
