# Corrigendum: β2-Adrenergic Receptor-Mediated HIF-1α Upregulation Mediates Blood Brain Barrier Damage in Acute Cerebral Ischemia

**DOI:** 10.3389/fnmol.2017.00392

**Published:** 2017-11-20

**Authors:** Yanyun Sun, Xi Chen, Xinyu Zhang, Xianzhi Shen, Mengwei Wang, Xiaona Wang, Wen-Cao Liu, Chun-Feng Liu, Jie Liu, Wenlan Liu, Xinchun Jin

**Affiliations:** ^1^Jiangsu Key Laboratory of Translational Research and Therapy for Neuro-Psycho-Diseases and Institute of Neuroscience, Department of Neurology, The Second Affiliated Hospital of Soochow University, Suzhou, China; ^2^School of Pharmacy, Key Laboratory of Molecular Pharmacology and Drug Evaluation, Yantai University, Ministry of Education, Yantai, China; ^3^The People's Hospital of Baoan Shenzhen, Shenzhen, China; ^4^Department of Emergency, Shanxi Provincial People's Hospital, Taiyuan, China; ^5^Translational Center for Stem Cell Research, Tongji Hospital, Stem Cell Research Center, Tongji University School of Medicine, Shanghai, China; ^6^The Central Laboratory, Shenzhen Second People's Hospital, Stem Cell Research Center, The First Affiliated Hospital of Shenzhen University, Shenzhen, China

**Keywords:** cerebral ischemia, HIF-1α, β2-AR, blood brain barrier, tight junction proteins, matrix metalloproteinase

There was a mistake in the section of “Acknowledgments” as published. The correct version appears below. The authors apologize for the mistake. This error does not change the scientific conclusions of the article in any way.

This work was supported by National Natural Science Foundation of China (81571149, 81671145), by Natural Science Foundation of Jiangsu Province of China (BK20151225), by Doctoral Fund of Ministry of Education of China (K521507713), by Suzhou Science and Technology Plan (SYS201518), by Jiangsu Provincial College of Natural Science research project (17KJB180012) and by Grants from Shenzhen Science and Technology Innovation Commission (JCYJ20150402152005623, GCZX2015050411225563, GJHZ20160301163900284), Guangdong Province Natural Science Foundation (2016A030313027). This work was also partly supported by Priority Academic Program Development of Jiangsu Higher Education Institutions of China.

## Conflict of interest statement

The authors declare that the research was conducted in the absence of any commercial or financial relationships that could be construed as a potential conflict of interest.

